# Looking beyond the thrombus: essentials of pulmonary artery imaging on CT

**DOI:** 10.1007/s13244-014-0340-6

**Published:** 2014-07-08

**Authors:** Mohammed M. Khadir, Apeksha Chaturvedi, Mike S. Nguyen, John C. Wandtke, Susan Hobbs, Abhishek Chaturvedi

**Affiliations:** Cardiothoracic Imaging Section, Department of Imaging Sciences, University of Rochester Medical Center, 601 Elmwood Ave, Rochester, NY 14642 USA

**Keywords:** Pulmonary artery, Congenital anomalies, Acquired anomalies, Embryology, Pulmonary embolus

## Abstract

**Background:**

Pulmonary arteries are not just affected by thrombus. Congenital and acquired conditions can also involve the pulmonary arteries. An awareness of these conditions is important for the radiologist interpreting chest computed tomography (CT).

**Methods:**

The anatomy of the pulmonary arteries was reviewed. CT and magnetic resonance (MR) acquisition protocols for imaging the pulmonary arteries were discussed. The imaging appearances of congenital and acquired anomalies involving the pulmonary arteries, using CT and other modalities, were presented.

**Results:**

Imaging features of congenital anomalies presented include pulmonary agenesis, partial pulmonary artery agenesis, patent ductus arteriosus, pulmonary artery sling, congenital pulmonary artery stenosis and coronary to pulmonary artery fistula. Acquired pulmonary artery anomalies discussed include arteritis, infected aneurysm and sarcoma. Pulmonary artery filling defects besides thromboembolism are also discussed, including foreign body emboli. Imaging features of bronchogenic carcinoma and mediastinal fibrosis demonstrating compression of the pulmonary arteries are presented, followed by a brief discussion of post repair appearance of the pulmonary arteries for congenital heart disease.

**Conclusions:**

Congenital and acquired pulmonary artery anomalies have a characteristic appearance on a variety of imaging modalities. An acquaintance with the imaging features of these anomalies is needed to avoid misinterpretation and reach the correct diagnosis.

*Teaching Points*

• *Discuss a variety of congenital and acquired anomalies of the pulmonary arteries*.

• *Discuss the imaging appearance of the presented congenital or acquired pulmonary artery anomalies*.

• *Describe CT and MR acquisition protocols for imaging the pulmonary arteries*.

• *Review the anatomy of the pulmonary arteries*.

## Introduction

**Fig. 1:**
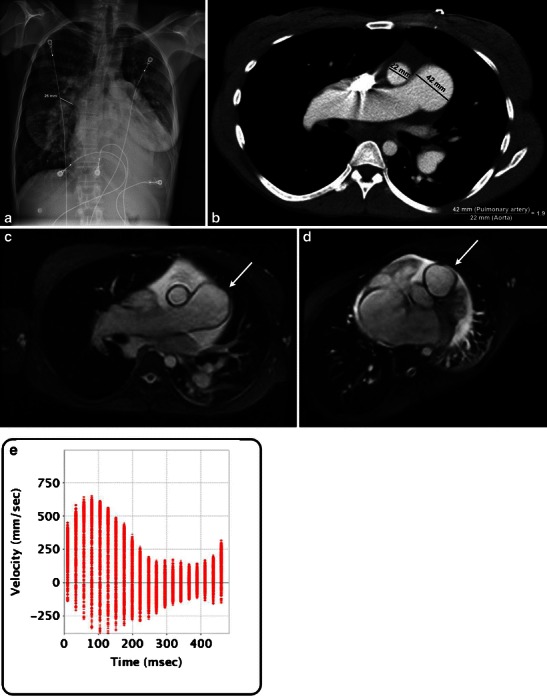
**a** Frontal radiograph of a patient with sinus venosus atrial septal defect demonstrates a dilated pulmonary artery, pulmonary oedema, left pleural effusion and cardiomegaly. The right lower lobe pulmonary artery measures 26 mm. **b** Contrast-enhanced axial CT image demonstrates an enlarged main pulmonary artery in the same patient. The pulmonary artery to aorta ratio is 1.9. **c** Axial steady state free precession images in the same patient demonstrate enlarged pulmonary artery (*arrow*). **d** Pulmonary artery distensibility (1.8 %) can be calculated using cine MR, by measuring the diastolic minimal (10.9 cm^2^) and systolic maximal (11.1 cm^2^) cross-sectional area obtained perpendicular to direction of blood flow. **e** Average velocity obtained with phase contrast MR in the main pulmonary artery is 11.6 cm/s

Often, the frontal chest radiograph provides the first clue to the presence of an abnormal pulmonary artery (Fig. 1a). If the pulmonary artery is enlarged, it presents with an enlarged contour of the vessel below the aortopulmonary window. Transverse diameter of the normal right interlobar artery from its lateral aspect to the intermediate bronchus is 15 mm in women and 16 mm in men. Computed tomography (CT) with intravenous contrast (Fig. 1b) provides more detail of the lumen, vessel wall and adjacent mediastinal structures. Greater anatomical detail is obtained with magnetic resonance (MR) imaging, allowing for improved evaluation of the vessel wall and quantification of flow (Fig. 1c). It also allows for pulmonary artery maximal and minimal cross-sectional area measurement to be made perpendicular to the axis of blood flow, useful in identifying distensibility (Fig. 1d) [1]. Positron emission tomography (PET)-CT is useful to evaluate for malignancy and arteritis. More invasive methods of imaging the pulmonary artery include intravascular ultrasound and catheter angiography.

In this article, we will briefly review the embryology and anatomy of the pulmonary arteries, followed by a discussion of the CT appearance of the common congenital anomalies and acquired conditions affecting the pulmonary arteries. For ease of discussion, the acquired entities will be categorised as those affecting the vessel wall, intraluminal abnormalities and extraluminal abnormalities. In addition, a brief discussion of imaging appearance in patients with repaired congenital heart diseases affecting the pulmonary arteries is also included.

## Embryology

**Fig. 2:**
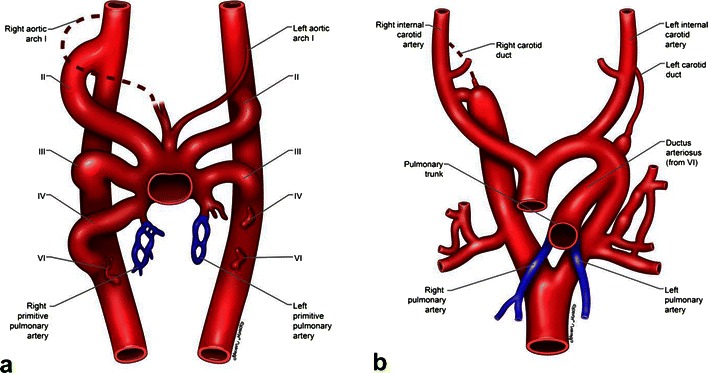
**a** Illustrations depicting the developing six paired aortic arches with the left and right dorsal aorta during early embryogenesis. **b** Further development leads to formation of right and left pulmonary arteries from the sixth aortic arches, primitive truncus arteriosus and adjacent arteries. Arrest in this normal development can lead to agenesis, partial agenesis, pulmonary sling, etc.

During the 4th-5th week of embryogenesis, the aortic sac gives rise to six paired arteries called the aortic arches, which will eventually develop into the mature aortic arch and other major vessels (Fig. 2). The arches originate from the aortic sac and terminate in the right and left dorsal aorta. The right sixth aortic arch persists as the proximal right pulmonary and the distal main pulmonary artery. The primitive truncus arteriosus forms the proximal main pulmonary artery. The left pulmonary artery and the distal right pulmonary artery develop from arteries arising from the adjacent lung buds and surrounding mesoderm [2].

## Anatomy

Two arterial circulations supply the lungs [3]. The bronchial circulation draws 1 % of systemic cardiac output and normally only supplies nutrients to the lungs. The primary circulation is the pulmonary arteries, which convey venous blood to the lungs from the heart. A pulmonary artery branch accompanies the bronchial tree and ends in capillary network within the alveolar wall [4]. The normal main pulmonary artery (MPA) divides into the right and left branches before it exits the pericardium. The left pulmonary artery (LPA) travels over the left mainstem bronchus before dividing into its two branches at the root of the left lung. The right pulmonary artery (RPA) continues from the MPA before diving into its two branches, the superior and inferior (interlobar) trunk, at the root of the right lung. The superior trunk supplies the right upper lobe with the interlobar trunk supplying the middle and lower lobes. The lobar branches divide into segmental and subsegmental arteries. Right middle lobe medial and lateral segmental arteries may arise as a common trunk from the interlobar artery or as separate branches. The right lower lobe artery first gives off an apical segmental branch and distal to this the right lower lobe artery is called the basal trunk. Lower lobe artery gives off the medial basal and anterior basal followed by the lateral and posterior basal segmental arteries. On the left, there is no truncus anterior, and the segmental branches originate directly from the LPA. For the left upper lobe and lingual arteries, there may be five to seven segmental branches. The superior segmental artery of the lower lobe arises from the left interlobar artery above the origin of lingular branches. Caudal to this, the left interlobar artery becomes basal trunk giving rise to lower lobe segmental branches [5]. The basal branches may be duplicated or triplicated [4].

On CT, the main pulmonary artery measures up to 28 mm, some studies have found 29 mm in men and 27 mm in women to be the upper limit for normal [3, 6]. A convenient method to evaluate for pulmonary artery enlargement is to determine whether the ratio of the main pulmonary artery to the ascending aorta (Fig. 1b) is greater than 0.9 [6].

Normal main pulmonary artery pressure ranges from 8 to 20 mmHg. In pulmonary hypertension (intraluminal pressure exceeding 25 mmHg at rest or 30 mmHg with exercise), frontal chest radiograph demonstrates a prominent pulmonary artery silhouette with dilated hilar vessels and diminished peripheral vascularity (Fig. 1a). Phase-contrast MR-derived mean average velocity <11.7 cm/s can help in detection of pulmonary hypertension (sensitivity 92.9 % and specificity 82.4 %) [7]. Pulmonary arterial transit times measured using time-resolved MR angiography can be used as a simple, non-invasive metric for detection of altered haemodynamics in pulmonary arterial hypertension [8]. Cine MR derived pulmonary artery distensibility of >10 % (systolic pulmonary artery area - diastolic pulmonary artery area ÷ systolic pulmonary artery area × 100) [1] is useful to evaluate pulmonary hypertensive patients who would respond to vasodilator therapy. In patients with Fontan circulation, pulmonary perfusion ratios are more accurately evaluated with phase contrast MR compared with lung perfusion scintigraphy [9].

## Acquisition protocols

### CT

Pulmonary CT angiography protocols have been evolving over the years for evaluating pulmonary embolus [10]. Adequate contrast opacification is critical for diagnostic quality, which depends upon patient weight, cardiac output, scan duration and contrast delivery protocol [11, 12]. Arterial enhancement depends on the amount of contrast delivered per unit of time (injection flow rate) and the injection duration, measured in seconds [13]. Suggested minimal luminal attenuation to see all acute and chronic pulmonary venous emboli (PE) is 93 and 211 HU respectively [14]. On a 64-detector CT, a mean pulmonary artery opacification of 250 HU could be achieved with 1.2 ml/kg of 350 mg I/ml injected at 4 ml/s [11]. Iodine flow rate of 1.6 g I/s has been suggested as optimal to reach the pulmonary artery enhancement of 300 HU [15]. The scan duration depends upon the scanner (16, 64, dual source, dual source high pitch, 256, 320 slice multidetector [MD] CT), which on a high pitch scanner this may be less than 2 seconds [16]. With a faster scanner, contrast volume can also be decreased by using a higher iodine concentration [12].

For CTA, a region of interest can be placed in the main pulmonary artery and a timing bolus or bolus tracking can be utilised to determine the time it takes for intravenously injected contrast to reach the pulmonary arteries [17]. Either of these techniques results in homogenous opacification and diagnostic image quality [18]. Contrast flow rate of at least 3 ml/s is associated with lower frequency of insufficient contrast enhancement during chest CT [19]. Flow rate of more than 4 ml/s using an 18-G cannula has been suggested for PE exams [20, 21] A lower volume of contrast and iodine dose can be administered using a higher concentration (350 mg iodine/ml vs 300 mg/ml) [22]. Wu et al. [23] have described a low contrast dose (30 ml) pulmonary 64-detector CT angiography technique without compromising diagnostic image quality. The duration of contrast administration is calculated as scan duration plus additional few seconds (6–8 s). This delay accounts for the interval between the scan trigger and the start of acquisition [12].

When evaluating for Fontan circulation, Park et al. [24] found that a 3-min delay time from the time of injection to be optimal for enhancement of the pulmonary arteries, irrespective of the intravenous route used for administration. Bolus tracking demonstrated a high failure rate in providing homogenous enhancement of the Fontan circulation and of the pulmonary arteries.

For all pulmonary CT angiography studies, a caudocranial direction of acquisition is recommended as it reduces the chances of having respiratory motion related artefacts [14]. At our institution, in-patients with normal (Stage 1, glomerular filtration rate [GFR mL/min/1.73 m2] = 90+) and mildly reduced renal function (Stage 2, GFR = 60–89) and no contraindication to CT contrast agent, contrast volume is determined from patient height, weight, age, sex, heart rate and scan duration using vendor-specified protocol (MEDRAD) with a timing bolus (test bolus of 20 ml contrast and 50 ml saline at 4 ml/s to find the time to peak in the main pulmonary artery is used to determine the scan delay, scan delay = time to peak in pulmonary artery + 9 s) [25]. The maximum allowed injection flow rate is 6 ml/s. In patients with moderately impaired renal function (stage 3 A, GFR = 45–60), bolus tracking with 75 ml of contrast at 4–5 ml/s is used. In patients with moderately reduced renal function (Stage 3 B, GFR = 30–44) 30 ml of contrast with bolus tracking from SVC, preferably on the 256 slice MDCT is used. Any contrast injection is avoided in patients with GFR less than 29 unless they are on haemodialysis. CT angiography protocol used at our institution is presented in Table 1.

**Table 1:** Pulmonary CT angiography protocol used at our institution

Indication	Contrast, flow rate	kVp^a^	mAs(AP scout)	Reconstructions	Comments
Congenital	Power or hand injection 3 ml/s, 50 ml contrast (300 mg I/ml), no saline chaser	Small = 80, medium = 100, large = 120 80–120	Tube current modulation	Axial: 3 × 2 mm, 2 × 1 mm Coronal: 3 × 2 Axial MIPS: 8 mm	25 s delay, Complete thorax
Pulmonary embolism	Dual head power injector 4–5 ml/s (350 mg/ml), + 50 ml saline chaser	80–140	Tube current modulation	Axial: 3 × 2 mm, 2 × 1 mm Coronal: 3×2 Axial MIPS: 8 mm	Weight-based contrast, Bolus track or timing bolus, Minimal post threshold delay
Pulmonary hypertension	Power or hand injection 2–3 ml/s, no saline chaser	80–140	Tube current modulation	Axial: 1 × 0.5 mm, 3 × 2 Coronal: 3 × 2 Axial MIPS: 8 mm	Low kVp, 50–75 ml contrast, Additional expiratory scans, HRCT recons
Pregnant patient	Dual head power injector 4–5 ml/s, + 50 ml saline chaser	80–100	Tube current modulation	Axial: 3 × 2 mm Coronal: 3 × 2 Axial MIPS: 8 mm	Low kVp, max. 75 ml contrast, *Z*-axis coverage: Aortic arch - diaphragm
Renal dysfunction	Dual head power injector 3–4 ml/s, + 50 ml saline chaser	80–120	Tube current modulation	Axial: 3 × 2 mm Coronal: 3 × 2 Axial MIPS: 8 mm	30–75 ml contrast, preferably on 256 MDCT, Trigger from SVC

^a^The kVp used depends on patient size: small = 80 (body mass index (BMI) <20 kg/m^2^), medium = 100 (BMI = 20–25), large = 120 (BMI = 25–30). Maximum tube current is determined by the frontal scout. For obese patients scan parameters based on scouts including kVp > 140

### MR

MR imaging for the diagnosis of pulmonary artery disease can be performed using high-field MR scanners (>1.5 T) [26]. It is indicated when cardiac function and flow needs to be evaluated, such as congenital heart disease, calculating intra/extra-cardiac shunts, right ventricle strain in PE and pulmonary hypertension. Non-contrast sequences used include a bright blood steady state free precession (SSFP), T2-weighted inversion recovery and T1 GRE (gradient echo). Post-contrast MR angiography is performed with extracellular gadolinium contrast agent injected at 0.1–0.2 mmol/kg. When evaluating for PE, a combination of MR angiography GRE and SSFP images have the highest sensitivity [27]. MR is the imaging modality of choice for evaluating the right ventricle size and function [28]. Contrast-enhanced MR angiography with gadolinium-based MRI contrast agent, using both high–spatial-resolution and high–temporal-resolution protocols (high–spatial-resolution contrast-enhanced MR angiography and time-resolved contrast-enhanced MR angiography), is an excellent non-invasive imaging tool for the evaluation of surgical cavopulmonary connections [29]. Pulmonary MR angiography should be considered as an alternative to CT angiography when iodine contrast injection or radiation is a significant matter [30]. It has been proposed that electrocardiograph (ECG)-gated and respiratory navigator-gated MR angiography at 3 T using a blood-pool contrast agent at 0.3 mmol/kg can deliver better image quality and vessel sharpness [31]. Although, gadolinium-based contrast agents are not recommended in patients with a GFR less than 30 or acute renal failure in patients with hepatorenal syndrome unless essential due to risk for nephrogenic systemic sclerosis [32]. Pulmonary MR angiography protocol used at our institution is presented in Table 2.

**Table 2:** Pulmonary MR angiography protocol used at our institution on a 1.5-T magnet

	Sequence type	Orientation	Slice thickness/gap (mm)	TE/TR (msec)	Flip angle (degrees)	Matrix	Field of view (mm)	Bandwidth (Khz)	NEX	Information acquired
Non-contrast	SSFP	Axial, coronal, ventricle short axis	4/0	1.4/3.4	45	200 × 160	350–420	125	0.75	Morphology, ventricle function
	T1	Axial, short axis	6/0	42	90	256	38	62.5	1	Morphology, characterise mass lesions, oedema
	T2	Axial, short axis	6/0	41/1,791	90	256 × 256	350	62.5	1	
	Phase Contrast	Perpendicular to pulmonary flow	8	2.7/5.6	25	192 × 128	350	31.25	1	Quantify pulmonary flow volume, peak-mean velocity, regurgitation
Contrast-enhanced MR angiography	MR angiography	Coronal	2.0	1.4/3.9	30	224 × 224	320–420	62.5	.5	Luminal assessment
	Time resolved	Coronal	2.6	1.2/3.2	38	256 × 192	40	62.5	0.5–0.75	
	3D GRE	Axial	4/–2	1.9/3.9	12	320 × 160	320–420	83.3	0.75	
	Delayed enhanced	Axial, short axis	8/0	1.3/5.3	20	224 × 192	35	22.7	1	Thrombus, vessel wall, inflammation/scar

### PET-CT

F-18 fluorodeoxyglucose (FDG) PET/CT is useful in identifying a pulmonary artery lesion as malignant if the luminal lesion has high FDG uptake [33] and is useful in preoperative evaluation [34]. It is also very useful in identifying active vasculitis in patients with pulmonary vasculitis such as Takayasu’s arteritis [35] and monitoring response to immunosuppressive treatment [36]. At our institution, a PET-CT for these indications is combined with a contrast-enhanced CT angiography of pulmonary arteries to better depict the vascular anatomy rather than a non-contrast CT for attenuation correction.

## Congenital

**Fig. 3:**
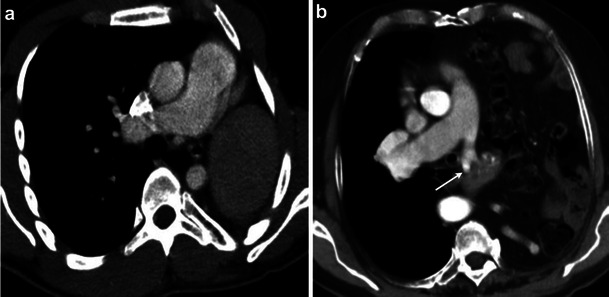
Contrast-enhanced CT image (**a**) shows complete agenesis of left lung and left pulmonary artery. The left hemithorax is smaller with mediastinal shift toward the left and the elevation of the left hemidiaphragm. The abdominal contents are seen in the left hemithorax. Contrast-enhanced axial CT image (**b**) demonstrates partial agenesis of the left pulmonary artery (*arrow*) with hypoplasia of left lung. There is no mediastinal shift, but the abdominal organs extend into the thorax

Unilateral pulmonary agenesis presents with unilateral absence of the lung and absence of the ipsilateral pulmonary artery and veins (Fig. 3a). The aetiology is unknown, although genetic factors, viral infections, folate and vitamin A deficiencies have been proposed as possible causes [37]. Newborns with this abnormality typically do not present with respiratory distress, but are likely to have other anomalies associated with the cardiovascular, musculoskeletal or gastrointestinal system. Later in life, patients may have poor lung function with recurrent respiratory infections. CT demonstrates decreased volume in the ipsilateral hemithorax, complete absence of lung parenchyma, agenesis of pulmonary artery and veins. There is elevation of hemidiaphragm and mediastinal shift to the affected side [38, 39].

Partial pulmonary artery agenesis involves an absence of the proximal portion or a rudimentary pulmonary artery. Blood flow to the ipsalateral lung is achieved through collaterals provided from the brachial arteries and transpleural branches of the thoracic arteries. Patients with the anomaly show an increased predisposition to dyspnea, recurrent respiratory infections and pulmonary haemorrhage. Chest radiographs demonstrate ipsilateral volume loss with hyperinflation of the contralateral side. CT illustrates (Fig. 3b) a rudimentary proximal vessel and hypoplastic lung. Transpleural collaterals can be seen as pleural thickening and subpleural parenchymal bands on the CT [3, 40].

**Fig. 4:**
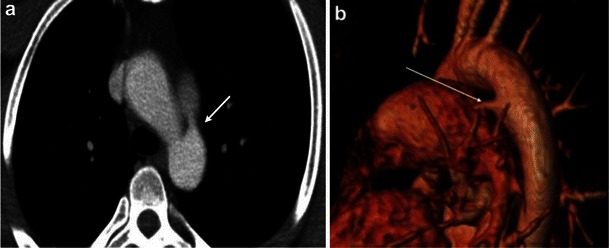
Contrast-enhanced axial CT image (**a**) and a volume rendered image (**b**) in a patient with patent ductus arteriosus (*PDA*) depicting the persistent communication between the pulmonary artery and descending aorta (*arrow*). The flow direction in the post-natal period is aorta to pulmonary artery as the pulmonary pressures decrease. This can lead to pulmonary hypertension, which on CT will present as enlarged pulmonary trunk as seen on the volume rendered image

The primitive left sixth aortic arch gives rise to the ductus arteriosus, which connects the descending thoracic aorta to the left pulmonary artery. Patent ductus arteriosus anomaly arises with persistent postnatal hypoxia, leading to failure of contraction of the ductus with formation of a continuous left to right shunt forms. A small shunt predisposes to endocarditis and a larger shunt causes haemodynamic derangement, eventually leading to Eisenmenger syndrome [41]. Symptomatic patients may present with dyspnea, tachycardia, a widened pulse pressure and a machinery-like continuous murmur. CT demonstrates dilated pulmonary artery, pruning of the peripheral compared with central pulmonary vasculature. Contrast-enhanced CT will identify the patent communication between the descending thoracic aorta and the pulmonary artery. Cardiac MR can be used to quantitate the left to right shunt (Fig. 4) [41, 42].

**Fig. 5:**
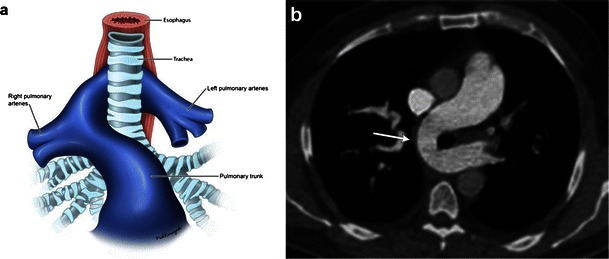
Illustration (**a**) and contrast-enhanced axial CT image (**b**) depicting the left pulmonary artery coursing between the trachea and oesophagus to reach the left pulmonary hilum. Patient’s symptoms correlate with the degree of upper airway obstruction present from narrowing of the trachea

Pulmonary artery sling presents when the left pulmonary artery arises from the posterior aspect of the right pulmonary artery before coursing between the trachea and oesophagus to reach the left hilum (Fig. 5). The sling around the distal trachea and right mainstem bronchus causes a variable amount of compression of these structures and may lead to stenosis of a long segment of the trachea. The amount of upper airway stenosis correlates to the degree of the patient’s symptoms. CT can accurately illustrate the anomaly. In addition, phase contrast MR may be used for quantification of pulmonary blood flow [3, 43]. Flow measurements are calculated from single slice phase contrast MR obtained perpendicular to MPA, RPA and LPA.

**Fig. 6:**
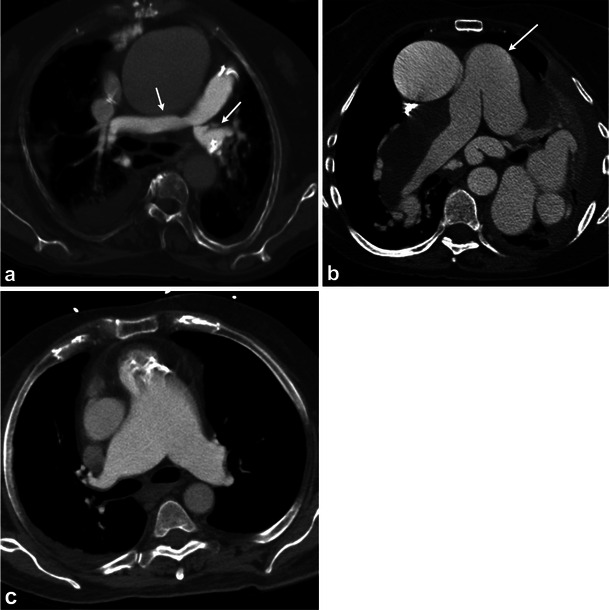
Contrast-enhanced axial CT image (**a**) from a patient with tetralogy of Fallot (*TOF*) and a prosthetic pulmonic valve demonstrates severe stenosis of the left and mild stenosis of the right pulmonary artery (*arrows*). In addition there is an ascending aortic aneurysm. In a different patient (**b**) with an unrepaired *TOF*, an aneurysm of the pulmonary trunk (*arrow*) formed with a chronic thrombus in the right and left pulmonary arteries. Also note the multiple dilated aortopulmonary collaterals. CT angiogram **c** performed with 30 ml of contrast in a patient with chronic renal failure and a prosthetic pulmonary valve demonstrates a main pulmonary artery aneurysm (measuring 44 mm)

Pulmonary artery stenosis leads to right ventricular outflow tract obstruction and can be secondary to a variety of congenital or acquired aetiologies. In tetralogy of Fallot (TOF), hemodynamic consequences depend largely on the degree of right ventricular outflow tract obstruction, including supravalvular narrowing, which has been reported in up to 50 % of patients [44]. Other congenital aetiologies for pulmonary artery stenosis include Williams syndrome, Alagille syndrome and congenital rubella [45]. Affected regions of the vessel demonstrate fibrous intimal proliferation with loss of elastic fibres, leading to varying degrees of stenosis. Post-stenotic segments may be dilated or aneurysmal and often is the first clue on radiographs. A pulmonary artery aneurysm is commonly defined as the pulmonary trunk measuring more than 4.5 cm and the right or left pulmonary artery measuring greater than 3 cm [46]. CT can demonstrate stenosis in the main and branch pulmonary arteries with dilated post-stenotic segment (Fig. 6) [47].

**Fig. 7:**
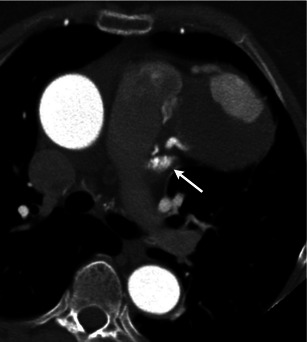
Contrast-enhanced axial CT image in systemic arterial phase demonstrates contrast blush within the pulmonary trunk emanating from a tubular enhancing structure along the left anterior descending coronary artery. Communication is noted between this and the pulmonary artery, suggesting a coronary to pulmonary artery fistula (*arrow*)

Coronary to pulmonary artery fistula is an anomaly that accounts for 15–30 % of all coronary artery fistulas [48]. The fistulous communication can either be congenital or acquired, as in the case of trauma, endovascular procedures and cardiac transplantation. In a few patients, a significant shunt can form, leading to congestive heart failure from volume overload or angina. Most reported cases have been incidentally detected during catheter angiography, but more recently CT angiography has been used to describe the features of the fistula. Both modalities demonstrate a direct communication between the two vessels. If the CT images are acquired in the systemic arterial phase, the only finding will be a contrast blush within the pulmonary artery (Fig. 7).

## Acquired

**Fig. 8:**
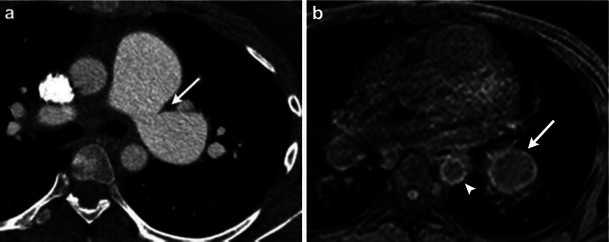
Contrast-enhanced axial CT image (**a**) in a 16-year-old patient with progressive dyspnea and absent left upper extremity pulse shows a focus of smooth narrowing and aneurysmal dilatation of the left main pulmonary artery (*arrow*). Late venous phase axial MR image from a 3D GRE acquisition (**b**) shows delayed enhancement of an aneurysmal left pulmonary artery branch (*arrow*). Also note the wall enhancement of descending thoracic aorta (*arrowhead*) consistent with vasculitis. These findings are suggestive of Takayasu arteritis

**Fig. 9:**
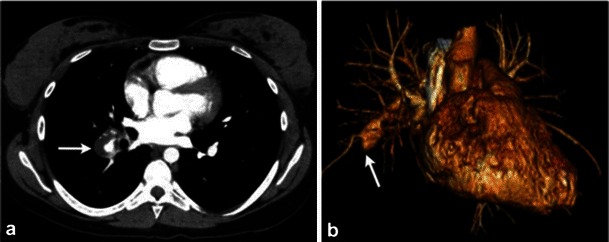
Contrast-enhanced axial CT (**a**) in a patient with Bechet’s disease demonstrate a focal aneurysm of the right lower lobe pulmonary artery with eccentric mural thrombus (*arrow*). Volume rendered image (**b**) better depicts the eccentric saccular aneurysm (*arrow*). This patient underwent right lower lobectomy for recurrent haemoptysis

Acquired diseases affecting the vessel wall include vasculitis, infected aneurysm and sarcoma. Takayasu arteritis is an idiopathic disorder producing granulomatous inflammation of the arterial wall. It involves the pulmonary artery is 50–80 % of cases. In early disease, the vessel wall may demonstrate enhancement and thickening, and in advanced disease, may demonstrate stenosis or occlusion [3, 49]. CT (Fig. 8) demonstrates wall enhancement, stenosis, ectasia or aneurysm of the affected vessels. Behcet disease is a chronic multisystem small vessel vasculitis that can cause aneurysmal dilatation of the pulmonary artery (Fig. 9). MR can be useful in demonstrating wall inflammation in either of these diseases [49].

**Fig. 10:**
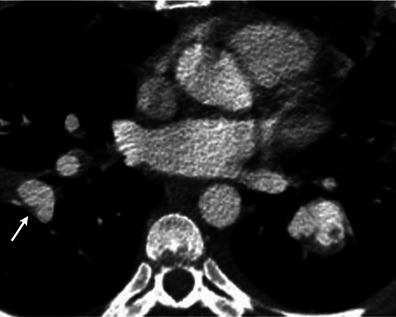
Contrast-enhanced axial CT image demonstrates aneurysmal formation with irregular thick walls in the segmental branches of right and left lower lobe pulmonary arteries (*arrow*) in this patient with a known infected aneurysm. These findings were new compared with prior chest CT

Infected (mycotic) aneurysm of the pulmonary artery can develop from haematogenous seeding of the infectious agent or continuous involvement from an adjacent source. *Staphylococcus*, *Streptococcus* and *Salmonella* are most often the infectious agents. CT angiogram (Fig. 10) is the modality of choice for evaluation of the infected aneurysm and demonstrates a lobulated vascular mass with an irregular wall arising from the vessel in question. In addition, the soft-tissues surrounding the aneurysm may demonstrate enhancement [50].

**Fig. 11:**
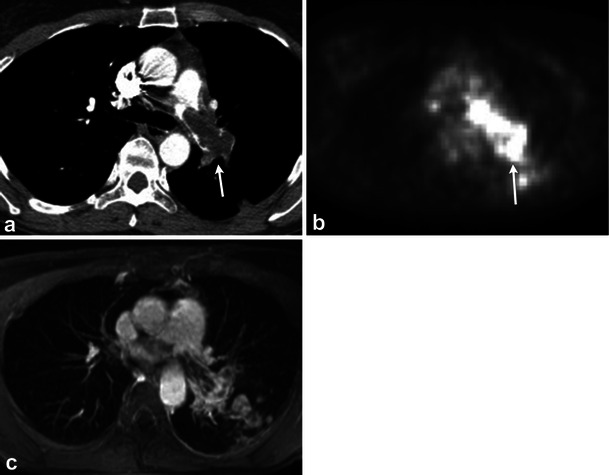
Contrast-enhanced axial CT image (**a**) demonstrates a large filling defect in the left pulmonary artery (*arrow*). The lesion remained stable after a course of anticoagulation, which raised the suspicion for a malignancy. Subsequently obtained FDG-PET (**b**) demonstrated the central part of this filling defect to be hypermetabolic, consistent with a primary pulmonary artery sarcoma. Gadolinium-enhanced cardiac MR (**c**) performed 60 s post contrast for preoperative evaluation demonstrates a lesion in the left pulmonary artery with an non-enhancing central portion, consistent with a bland thrombus, and an enhancing component in the pulmonary arteries and left lower lobe, suggestive of a tumour

Pulmonary artery sarcoma arises from the mesenchymal cells of the intima. On initial evaluation, the entity is often misdiagnosed as a pulmonary embolism because of similar presentation. The two entities can be differentiated using a contrast-enhanced CT by evaluating for a low-attenuation filling defect occupying the entire lumen and leading to expansion of the artery or with extraluminal tumour extension (Fig. 11a) [3]. FDG-PET shows the sarcoma to have higher metabolic activity than blood pool [33] (Fig. 11b).

**Fig. 12:**
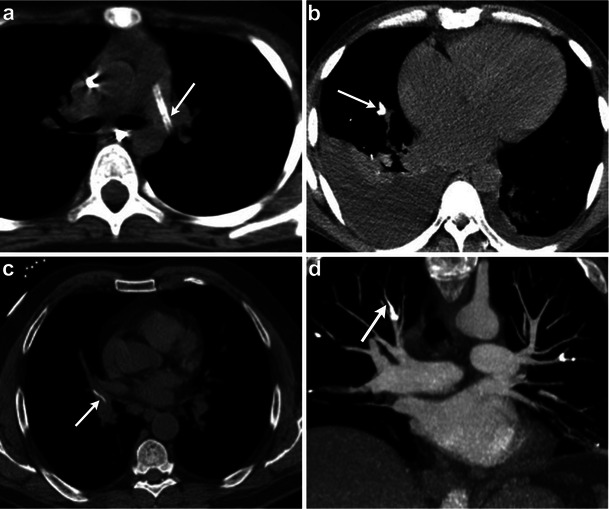
Different patients with non-thrombotic emboli to the pulmonary arteries: catheter fragment (**a**), non-target emboli from *N*-butyl-2-cyanoacrylate injection of gastric varices (**b**), inferior vena cava filter prong (**c**) and bone cement for vertebropasty (**d**)

The majority of intraluminal filling defects of the pulmonary artery are secondary to pulmonary thromboembolism. Several malignancies, including breast and colorectal carcinoma, metastasise to the lungs by the way of the pulmonary arteries. Intraluminal enhancing filling defects of the pulmonary arteries in these patients may represent metastases. In addition, the pulmonary arteries maybe the site for non-thrombotic emboli, such as non-target embolisation of intravascular glue, broken embolised fragments of an IVC filter or vertebroplasty cement (Fig. 12).

**Fig. 13:**
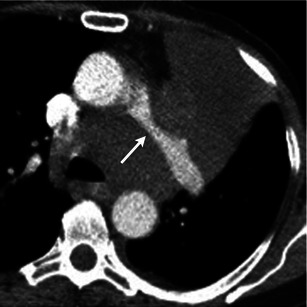
Contrast-enhanced axial CT image in a patient with left hilar lung cancer demonstrates the left main pulmonary artery being completely encased and narrowed by the left upper lobe mass (*arrow*), which also extends into the mediastinum

**Fig. 14:**
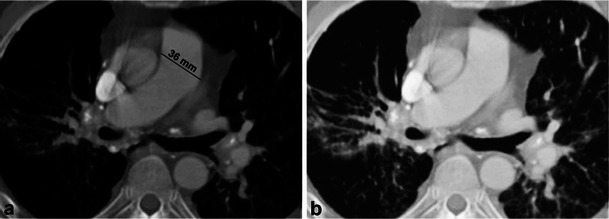
Axial CT images in a patient with prior histoplasmosis demonstrates an enlarged pulmonary trunk (36 mm). The proximal right and left pulmonary arteries are normal in calibre but taper and are severely narrowed at the level of hila. In addition, there are calcified mediastinal lymph nodes, calcified pulmonary granulomas and interlobular interstitial thickening. These findings represent fibrosing mediastinitis

The pulmonary artery can also be affected by extrinsic processes. Luminal narrowing of the pulmonary artery may be due to extrinsic compression from bronchogenic carcinoma (Fig. 13), lymphadenopathy or mediastinal fibrosis encasing the vessel [3]. Pulmonary artery dilatation can be seen with pulmonary hypertension, which can be secondary to a pulmonary parenchymal disease. CT is essential in evaluating the lung parenchyma and, in addition, will demonstrate pulmonary artery diameter greater than 28 mm or a pulmonary artery to ascending aorta transverse diameter ratio greater than 0.9 [6, 51]. Granulomatous fibrosing mediastinitis is an infiltrative disorder that results from excessive fibrosis in the mediastinum, usually a sequela of histoplasmosis (Fig. 14). It can result in encasement of the mediastinal viscera with narrowing of the vessels, airway, and other mediastinal structures [52].

**Fig. 15:**
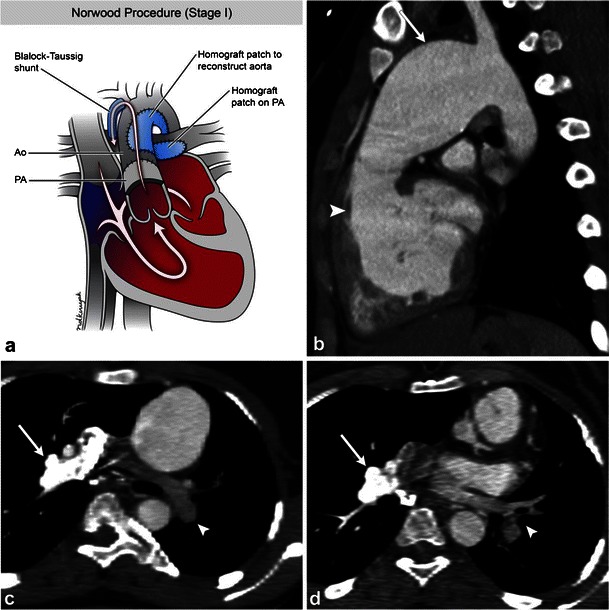
Illustration (**a**) demonstrates stage 1, the Norwood procedure, for correcting hypoplastic left heart syndrome with the creation of a neoaorta from the pulmonary artery. Post-repair images (**b**) have a characteristic appearance with a rudimentary proximal ascending aorta and the proximal main pulmonary artery (*arrow*) reconstituting flow to the distal ascending aorta (*arrowhead*). During the procedure, the pulmonary trunk is ligated and the pulmonary arterial flow is re-established from either the subclavian artery or the brachiocephalic trunk. After completion of stage 3 (**c**, **d**), by attaching the inferior vena cava to the right pulmonary artery, the Fontan procedure, complete systemic venous flow is directed through the right pulmonary artery into the lungs. Note the right pulmonary artery (*arrow*) shows higher attenuation secondary to the contrast injection from the right arm veins compared with the left pulmonary artery (*arrowhead*) which has lower attenuation due to blood flow from the inferior vena cava

Corrective surgical procedures for congenital cardiovascular diseases which affect the pulmonary arteries result in a characteristic appearance. Cavopulmonary shunts or Fontan circulation are used to treat infants with single effective ventricle (tricuspid/pulmonary atresia, hypoplastic left heart/hypoplastic right heart syndrome). The venous return is diverted to the pulmonary arteries bypassing the morphological right ventricle. The Norwood procedure is used to correct hypoplastic left heart syndrome, which is frequently associated with hypoplasia of the ascending aorta. Stage 1 involves creating a neoaorta from the proximal main pulmonary artery, which is connected to the ascending aorta (Figs. 15a and b). The right subclavian artery or the brachiocephalic trunk is then connected to the right pulmonary artery to provide blood flow to the lungs. Stage 2 of the procedure creates a Glenn shunt, a superior cavopulmonary shunt from an end-to-end anastomosis between the superior vena cava and right pulmonary artery, thus directing systemic venous flow directly to the lungs. Stage 3 creates a total cavopulmonary connection by attaching the inferior vena cava to the right pulmonary artery, referred to as a Fontan procedure [53, 54] (Figs. 15c and d). Contrast timing during pulmonary CT angiography is critical in such patients to when evaluating for a suspected stenosis or PE.

**Fig. 16:**
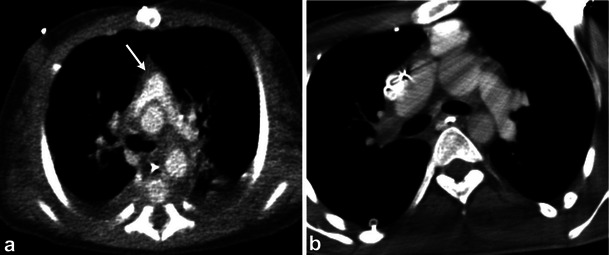
Contrast enhanced axial CT images in a patient with transposition of great vessels demonstrates the characteristic appearance post-arterial switch. The pulmonary arteries (*arrow*) are positioned anterior to the aorta with the left and right main branches draping around the aorta. There is a higher incidence of pulmonary artery stenosis in these patients

An arterial switch is performed for treating transposition of great arteries. It results in a characteristic appearance of the main pulmonary artery situated anterior to the ascending aorta with the right and left pulmonary arteries draped around the aorta. This repair can be associated with narrowing of the pulmonary arteries (Fig. 16).

## Conclusion

Congenital and acquired pulmonary artery anomalies have a characteristic appearance on a variety of imaging modalities. Even though imaging findings on CT were mainly discussed, the interpreting radiologist needs to be familiar with findings of these entities on a spectrum of imaging modalities to avoid misinterpretation and reach the correct diagnosis.

## Appendix

**Table 3:** Maximum contrast volume for CT angiography using power injector is based on patient weight (iodine concentration of 350 mg/ml)

Weight (kg)	Contrast volume (ml)
40	69
45	77
50	86
55	95
60	103
65	112
70	120
75	129
80	137
85	143
90	143
